# Energy Drink Consumption Practices of Young People in Bahrain

**DOI:** 10.5195/cajgh.2015.216

**Published:** 2016-03-17

**Authors:** Maryam M. Nassaif, Ghufran J. J. Alobed, Noor A. A. Alaam, Abdulla N. Alderrazi, Muyssar S. Awdhalla, Asokan G. Vaithinathan

**Affiliations:** College of Health Sciences, University of Bahrain, Bahrain

**Keywords:** energy drinks, health effects, young people, health promotion

## Abstract

**Background:**

Energy drink (ED) consumption is becoming increasingly popular among young Bahrainis, who may be unaware of the health risks associated with ED consumption. To date, there have been few publications on the consumption of ED in Bahrain, particularly among adolescents. This study seeks to fill a gap in the literature on energy drink consumption practices of Bahraini adolescents.

**Methods:**

Data were collected using a previously established European Food Safety Authority questionnaire. Cross-sectional analyses were conducted on a convenience sample of 262 Bahraini students aged 10 to 18 years.

**Results:**

Most participants consumed energy drinks 2 to 3 times per week and consumed two or more cans at a time. Eighty percent of partcipants preferred energy drinks with sugar. Participants in the older age group and higher educational level consumed more ED. The majority (57%) consumed ED at home with friends as part of socialization. Notably, 60% of the parents of the respondents have not consumed energy drinks. Prominent reasons for consumption of energy drinks included: taste (40%), energy (30%), stay awake (13%), augment concentration (4%), and enhance sports performance (6%).

**Conclusion:**

Energy drink consumption is a popular socialization activity among adolescents of Bahrain. The potential health risks necessitates the need for novel health promotion strategies and advocacy efforts for healthy hydration practices.

Energy drink(s) (ED) refers to beverages that contain caffeine in combination with other ingredients such as taurine, guarana, and B vitamins, with claims to provide its consumers with extra energy.^1^ Over 140 countries report ED consumption, with children, adolescents, and young adults representing half of the consumers of ED.^2^ The prevalence of ED consumption varies by age group and size. Regionally, ED are largely consumed in the Middle East and in the Western World. For instance, 55% of male and 26% of female students in the University of Dammam consumed ED,^3^ and Saudi Arabia was ranked among the top ten ED consuming countries.^4^ In 2013, the Central Information Organization of Bahrain reported that the consumption of ED in Bahrain was 174 per 1,000 persons.^5^ In the USA, about 1 in 9 youths received counseling discouraging ED consumption from a health care professional.^6^ The prevalence of ED consumption in Europe was 68%, varying from 48% in Greece to 82% in the Czech Republic, and mostly observed in the age group of 15–18 years (73%).^7^ On average, adolescents consume 2.1 L of ED per month in Europe.^7^ An Australian report revealed that 48% of young adults consumed ED at least once a month with an average intake of 1–2 cans per day.^8^

EDs act as non-nutritive stimulants with purported ergogenic or performance-enhancing effects. Caffeine, taurine, D-glucorono-y-lactone, and sugar are the main ingredients. Other products found in ED are vitamins, L-carnitine, and extracts such as guarana (1 gm of guarana equates 40 to 80 mg of caffeine), ginkgo biloba, bitter orange, and ginseng.^2^ The intended effects of ED are to provide sustenance, endurance, concentration, and enhanced performance. Manufacturers of ED target their sales to students, athletes, and people in professions that warrant sustained alertness.^9^ Popularity of ED among teenagers is associated with risk taking.^10^

Studies have shown that moderate caffeine consumption (<400mg/day) is not associated with the adverse effects of caffeine such as general toxicity, cardiovascular effects, effects on bone status, and calcium balance;^11^ however, the amounts of caffeine in ED far exceed that of safety limits.^11^ According to the World Health Organization (WHO) 2015 sugar intake for adults and children guidelines,^12^ the suggested limit of sugar consumption for adults of normal BMI is 25g/day (6 teaspoons). In general, sugar content in ED ranges from 21 g to 34 g per 8 oz. Daily consumers of two or three cans of ED could be ingesting 4 to 6 times the maximum recommended daily intake of sugar, which poses a risk for obesity and dental problems. 13

Common adverse effects of ED that have been documented are dizziness, inability to focus, nervousness, gastrointestinal upset, and insomnia.^14^ In rare situations, anxiety, seizure, increased heart rate, dehydration, acute mania, stroke, and behavioral problems like fighting and addiction have been observed.^14^ Potential problems associated with ED consumption in children and adolescents include cardiovascular effects and eating disorders. A systematic review suggests using caution in consuming ED, even though long term studies with health effects follow up were lacking.^14^ The prevalence of non-communicable diseases is similar to other developed economies according to the National Non-Communicable Diseases (NNCD) risk factor survey carried out in Bahrain,^15^ thus nutritional factors are important to investigate.

As of 2016, no reports have been published on the consumption practices of ED in Bahrain. Therefore, this study was undertaken to explore energy drink consumption practices of Bahraini adolescents.

## Methods

Data collection for this cross-sectional study was carried out by study investigators using a convenience sampling approach from public places that students frequently visit (i.e. parks and malls of Bahrain during the weekends). Our study sample included Bahraini adolescent students aged 10 to 18 years of both genders.

To be 95% confident that the true value of the estimate will be within 5 percentage points of the prevalence of 17%,^5^ the required sample size was calculated to be 217 in order to achieve the desired level of accuracy. The final number of participants recruited was 262. In the initial phase of data collection, participants were asked whether they had ever consumed ED. Those that answered yes were given a structured, self-report questionnaire. The questionnaire was modified from a previously established questionnaire for gathering consumption data on specific consumer groups of energy drinks by the European Food Safety Authority (EFSA).^16^ The modifications introduced were designed to make the questionnaire more suitable and compatible with the Bahraini society regarding demographic, economic, and socio-cultural aspects. Broadly, the questionnaire had two sections: 1) demographic data (i.e., age, gender, and current education level); 2) energy drink consumption data (i.e., consumption frequency, consumption amount, place of consumption, reasons for consumption, choice of sugar or sugar free, preferred brand, and parental consumption). For affirming face and content validity, the questionnaire was scrutinized by a panel of experts from the College of Health Sciences and Nutrition in Bahrain. The questionnaire was translated into Arabic and back translated to English to ensure there were no translation errors. Before beginning the study, the questionnaire was piloted with 30 participants to determine feasibility.

The study was approved by the institutional research committee of the College of Health Sciences, Bahrain. After explaining the study purpose, a written informed consent was obtained from all of the participants. Confidentiality of the participants and protection of data gathered was ensured by using study codes on data documents and removing all identifiable information.

### Data Analysis

The data from the questionnaires were cleaned, coded, and entered in Excel (Microsoft, Redmond, WA, USA) and then exported to SPSS version 21 (SPSS Inc, Chicago, Illinois, USA) for analysis. Descriptive statistics were used to analyze baseline participant characteristics. Chi-square tests were used to compare the ED consumption patterns and baseline characteristics of the participants.

## Results

The median age of the participants was 16 years (range: 10–18), which was not normally distributed. The majority of participants were enrolled in secondary education institutions (grades 10 to 12) and 51% were male (Table 1).

Analyzing the consumption practices, habitual consumption of ED was significantly higher in those aged 16 to 18 compared to those aged 10 to 12 or 13 to 15 (*p*<0.01), and a similar significant difference was observed in the group with higher levels of education compared to groups with two other levels of education (*p*<0.03). Sixty one percent of participants had initiated ED consumption in the past year. Fifty seven percent declared that they consumed ED at home and with friends as a part of socialization adjoining sedentary high screen media use such as watching TV or playing video games; less than 10% consumed ED during physical activities and sports or in public places. Frequency of ED consumption varied in the sample (Table 2). Among the respondents, one third consumed ED two to three days a week, and one fifth consumed ED less than once per month. Over 80% of respondants preferred a portion size of 250 ml more than any other available choice of portions. In an average month of the past year, 34% of the respondents had 2–4 cans and 23% of the respondents had 5–10 cans. In a single session of ED consumption over the last year 51% consumed either two or more cans.

Table 3 describes respondents’ key reasons for consumption of ED. Among them, 40% liked the taste of the drink, and 30% thought that ED provides the needed energy. Other reasons included: stay awake (13%), augment concentration (4%), and enhance sports performance (6%). The top three popular brands consumed by the participants of this study were Red Bull, Boom Boom, and Bison. Over 80% preferred ED with sugar over sugar free ED, and no difference in the preference between the age groups, gender or educational levels was found. Among the parents of the respondents, 40% have consumed ED.

## Discussion

Most participants consumed ED 2 to 3 times per week, two or more cans at a time, and primarily preferred ED with sugar. Older participants tended to consume more ED. Many consumed ED as a part of socialization.

This study supports the idea that ED marketing strategies have reached young people in Bahrain, since the majority of the participants consumed two or more cans in a single session 2 to 3 times a week, which is similar to the frequency of ED consumption reported from Italy.^17^ This study showed that in our sample, higher intake of ED was found in the older age group with a higher educational level, which may be due to independent money spending capacity and the ability to buy ED without parental supervision. The majority admitted to consuming ED at home and with friends as part of socialization. The primary reasons for consumption of ED were for the taste of the drink and the energy need. These findings corroborate the reasons adolescents consume ED given in a report^18^ from neighboring Saudi Arabia Findings of our study contradict the belief that the intended effect of ED use is endurance and physical activity. Less than 10% of the respondents reported that they consumed ED during physical activities or sports.

The majority of the respondents were habitual consumers of ED and preferred ED with sugar, indicating that this practice may also be associated with higher consumption of other sugar-sweetened beverages. High consumption of sweetened ED without physical activity, along with sedentary high screen media use among young people, are risk factors for non-communicable diseases, such as cardiovascular disease and obesity. This finding can be cautiously corroborated by the NNCD survey in Bahrain. Almost two thirds of adults in Bahrain were reported to be consumers of high calorie sugar-sweetened beverages and less than 3% used artificial sweeteners instead of sugar.^5^ NNCD has also revealed that the overall prevalence of overweight was 33%, obesity was 36%, diabetes mellitus was 14%, and hypercholesterolemia was 41%.^15^ Considering that over 60% of parents did not consume ED, and generalizing the prevalence of non-communicable diseases by the NNCD survey to our study, there is a trend of unhealthy consumption practices of ED by the current generation of young people in Bahrain, and with the anticipated demographic shift of these young people to adults, the burden of non-communicable diseases in Bahrain is expected to escalate over the reported NNCD survey prevalence rates. At the same time, it is possible that ED consumption in various situations may serve as a marker for other unhealthy behaviors among young people.^19^

Adolescents may not be fully capable of understanding complex concepts of behavior and health consequences, and their behavior patterns are different from children and adults. The successful transition period from childhood to adulthood of young people depends on the support of families, communities, schools, and health services. These support systems have the responsibility to promote their development and intervene effectively when problems arise.^20^

Increasing popularity of ED, particularly with adolescents, has been observed since their introduction at the end of the last century. It has been suggested that some young adults consume ED for their perceived physiologic benefits, unaware of the ingredients in ED and the associated health risks.^21^ The American Academy of Pediatrics^22^ suggests that ED pose potential health risks to children and young adults primarily due to the stimulant content. Often, young people do not distinguish ED from sports drinks. Therefore, this exploratory study was conducted to investigate the consumption practices of ED among adolescents in Bahrain.

This preliminary study has a limitation. The convenience sampling method used in this study of over 200 individuals may not be representative of the general Bahraini population. Regardless of this limitation, our study employed an EFSA validated survey tool with reliable measures to examine practices of ED consumption among young people of Bahrain. We recommend larger cross-sectional and logitudinal studies in the future to address this limitation.

## Conclusion

This study has shown that the ED consumption is a popular, high frequency socialization tool used among young people of Bahrain. The potential health risks that high ED consumption may cause necessitates the need for novel health promotion strategies. To prevent consumption at home, where the majority reported consuming ED, parents should be educated about the potential consequences associated with ED consumption and promote healthy habits of hydration. Fundamental to the health promotion campaign is instituting nutrition educational programs in educational institutions. The encouragement of the consumption of beverages with nutritive value, or water, and restricting the sale and use of ED on the institutional premises could curtail ED consumption. Taking the experience from the health promotions of the Saudi government^23^ and the active health promotion campaign in Bahrain, health regulatory authorities may consider warning labels on the containers of ED, and regulation of advertisements of ED similar to alcohol and tobacco.

## Figures and Tables

**Table 1:** Qualitative demographic characteristics of participants

Variables	N (%)
Age (in years)	
10–12	38 (14.5)
13–15	90 (34.4)
16–18	134 (51.1)
Gender	
Male	133 (50.8)
Female	129 (49.2)
Education	
Primary (Grades 1–6)	31 (11.8)
Intermediate (Grades 7–9)	81 (30.9)
Secondary (Grades 10–12)	150 (57.3)

**Table 2:** Frequency and volume of energy drink consumption

	N(%)
Frequency of energy drinks consumed per month	
Rarely	42 (22.8)
Once-twice a month	28 (15.2)
Once per week	19 (10.3)
2–3 days per week	53 (28.8)
4–5 days per week	19 (10.3)
Everyday	23 (12.5)
Consumption of energy drinks by volume	
250 ml can	149 (81.0)
355 ml can	28 (15.2)
Others	7 (3.8)
Frequency of energy drinks consumed per month	
1 or less	40 (21.7)
2 to 4 cans	63 (34.2)
5 to 10 cans	42 (22.8)
11 to 20 cans	24 (13.0)
>20 cans	15 (8.2)
Number of cans of energy drinks consumed in a single session	
1 Can	88 (47.8)
2 Cans	59 (32.1)
3 Cans	20 (10.9)
4 Cans	7 (3.8)
>4 Cans	10 (5.4)

**Table 3:** Reasons for energy drink consumption

	N(%)
Need energy (in general)	54 (30.0)
Stay awake	24 (13.3)
I like their taste	72 (40.0)
Concentration augmenting (Studying/Working)	8 (4.4)
Enhance sport performance	11 (6.1)
Effect of promotions and advertisements	3 (1.7)
Stimulate my metabolism	1 (0.6)
Others	7 (3.9)
